# Impacts of invasive annuals on soil carbon and nitrogen storage in southern California depend on the identity of the invader

**DOI:** 10.1002/ece3.5104

**Published:** 2019-04-01

**Authors:** Tal Caspi, Lauren A. Hartz, Alondra E. Soto Villa, Jenna A. Loesberg, Colin R. Robins, Wallace M. Meyer

**Affiliations:** ^1^ W.M. Keck Science Department, Claremont McKenna Pitzer, and Scripps Colleges Claremont California; ^2^ Department of Biology Pomona College Claremont California

**Keywords:** carbon sequestration, climate change, grassland, nitrogen availability, nutrient storage, sage scrub

## Abstract

Non‐native plant invasions can alter nutrient cycling processes and contribute to global climate change. In southern California, California sage scrub (hereafter sage scrub), a native shrub‐dominated habitat type in lowland areas, has decreased to <10% of its original distribution. Postdisturbance type‐conversion to non‐native annual grassland, and increasingly to mustard‐dominated invasive forbland, is a key contributor to sage scrub loss. To better understand how type‐conversion by common invasive annuals impacts carbon (C) and nitrogen (N) storage in surface soils, we examined how the identity of the invader (non‐native grasses, *Bromus* spp.; and non‐native forbs, *Brassica nigra*), microbial concentrations, and soil properties interact to influence soil nutrient storage in adjacent native and invasive habitat types at nine sites along a coast to inland gradient. We found that the impact of type‐conversion on nutrient storage was contingent upon the invasive plant type. Sage scrub soils stored more C and N than non‐native grasslands, whereas non‐native forblands had nutrient storage similar to or higher than sage scrub. We calculate that >940 t C km^−2^ and >60 t N km^−2^ are lost when sage scrub converts to grass‐dominated habitat, demonstrating that grass invasions are significant regional contributors to greenhouse gas emissions. We found that sites with greater total C and N storage were associated with high cation exchange capacities and bacterial concentrations. Non‐native grassland habitat type was a predictor of lower total C, and soil pH, which was greatest in invasive habitats, was a predictor of lower total N. We demonstrate that modeling regional nutrient storage requires accurate classification of habitat type and fine‐scale quantification of cation exchange capacity, pH, and bacterial abundance. Our results provide evidence that efforts to restore and conserve sage scrub enhance nutrient storage, a key ecosystem service reducing atmospheric CO_2_ concentrations.

## 1 INTRODUCTION

The impacts of non‐native plant invasion on nutrient cycling in soil are understudied, though the effects can be dramatic (Bradley, Houghton, Mustard, & Hamburg, 2006; Ehrenfeld, 2003; Jobbágy & Jackson, 2000). In a broad survey of how plant invasions change soil properties and ecosystem processes, Ehrenfeld (2003) demonstrated that invasion impacts are not unidirectional. Invasions may increase, decrease, or cause no significant changes to soil C and N concentrations, and differences between native and non‐native communities for C cycling processes may not reflect changes to N cycling processes. Because non‐native plant invasions can dramatically, and often unpredictably, modify soils biologically and chemically (Dickens, Allen, Santiago, & Crowley, 2013; Ehrenfeld & Scott, 2001), how plant invasions influence nutrient cycling processes depends on the identity of the invader and ecological context.

Non‐native grasses are widespread, successful invaders that effectively alter ecosystem functions, including C and N cycling (D'Antonio & Vitousek, 1992; Mack & D'Antonio, 1998). Non‐native grass invasion is especially severe in arid and semiarid environments like southern California, where European *Bromus* species have caused tremendous changes in ecosystem properties (D'Antonio & Vitousek, 1992; Wheeler et al., 2016). Increases in N deposition and disturbance regimes combined with habitat fragmentation have facilitated widespread *Bromus* spp. distributions (Cox, Preston, Johnson, Minnich, & Allen, 2014; Kimball, Goulden, Suding, & Parker, 2014; Talluto & Suding, 2008). Grass invasion also reduces fire return intervals, creating a positive feedback loop that facilitates further invasion because grasses are often the first colonizers after a disturbance (Brooks et al., 2004; D'Antonio & Vitousek, 1992; Fleming, Diffendorfer, & Zedler, 2009; Keeley, Baer‐Keeley, & Fotheringham, 2005; Syphard, Clarke, & Franklin, 2007). Throughout low‐elevation southern California, the success of invasive grasses has culminated in the widespread replacement of the native California sage scrub ecosystem (hereafter sage scrub) by non‐native grass species in a process known as type‐conversion (Cox et al., 2014; Talluto & Suding, 2008). Since the 1930s, sage scrub not lost to development has declined by nearly 50% mainly due to type‐conversion to non‐native grassland (Riordan & Rundel, 2014; Rundel, 2007; Talluto & Suding, 2008).

Research by Wheeler et al. (2016) and Caspi et al. (2018) in southern California suggests that type‐conversion has negatively impacted soil C storage in the region. Both studies found decreased soil C concentrations in non‐native grasslands compared to adjacent sage scrub habitats. While Wheeler et al. (2016) found decreased N in soil under type‐converted non‐native grasslands, Caspi et al. (2018) did not find significant soil N differences between habitat types, suggesting that N storage may be particularly influenced by interannual variation in environmental conditions. Caspi et al. (2018) also suggest that C storage capacities vary along spatial gradients, with coastal sites having higher storage ability than inland sites. These findings pose serious implications for regional carbon storage ability under increasing non‐native grass invasion, but our understanding of the mechanisms driving these processes remains limited. For example, findings from a single coastal site and two inland sites may not be representative of the broader region. Specifically, it is unclear whether all coastal sites store more C than inland sites, and whether patterns between habitats are consistent among seasons and years and across multiple sites.

Increasingly, *Brassica nigra*(black mustard) is becoming a widespread invasive, often being the dominant species following a disturbance instead of grasses (Bell & Muller, 1973; Keeley, 2001; Lambert, D'Antonio, & Dudley, 2010). Grasses can be excluded from *Brassica* stands as water‐soluble allelochemicals emitted as secondary plant products are known to inhibit the establishment of grass species (Bell & Muller, 1973; Muller, 1969; Turk & Tawaha, 2003). These toxins emitted by *Brassica* plants also degrade mycorrhizal fungi symbioses, significantly altering microbial assemblages in the soil (Pakpour & Klironomos, 2015). Because of the competitive exclusion of grasses and alteration of the soil microbial community, soil C and N storage in areas dominated by invasive mustards could differ from areas dominated by *Bromus*species. Despite this possibility, previous studies investigating type‐conversion have not differentiated between invasive cover dominated by non‐native forbs like *Brassica*and invasive habitat composed mostly of non‐native grasses (Caspi et al., 2018; Matsuda et al., 2011; Suarez, Bolger, & Case, 1998; Talluto & Suding, 2008).

While type‐conversion is a key process in terrestrial accounting of soil C and N, other factors must also be included in regional modeling efforts. Variability in soil taxonomy, structure, and soil density influences nutrient storage capacity across the landscape and can have significant effects across small distances (Caspi et al., 2018). Additionally, soil microbes play critical roles as drivers of biogeochemical cycling (Fierer et al., 2012), and differences between microbial assemblages between sage scrub and non‐native grassland habitats may have significant functional implications (Caspi et al., 2018; Dickens et al., 2013; Hawkes, Wren, Herman, & Firestone, 2005; Sigüenza, Crowley, & Allen, 2006). While some analyses incorporate microbial species diversity, describing microbial abundances alone may be informative. For example, the dominance of bacteria or fungi correlates with lowered or enhanced storage capacity, respectively (Allison, Miller, Jastrow, Matamala, & Zak, 2005; Manning, 2012; Wardle, 2002). Among other factors, C and N in surface soil horizons are controlled by soil mineralogy, pH, and organic matter (OM) inputs from plant growth, and are balanced by respiration of the soil microbial community (Houghton, 2007). Inputs, pools, and losses of C and N in terrestrial ecosystems are controlled by these soil and biotic processes (von Sperber et al., 2017). Complex links among factors must be considered to understand invasive species' impacts and to accurately account for soil C and N storage. As such, better predictive modeling of the consequences of widespread type‐conversion on terrestrial C and N budgets requires addressing the role of these multiple factors across spatial and temporal gradients.

**Figure 1:**
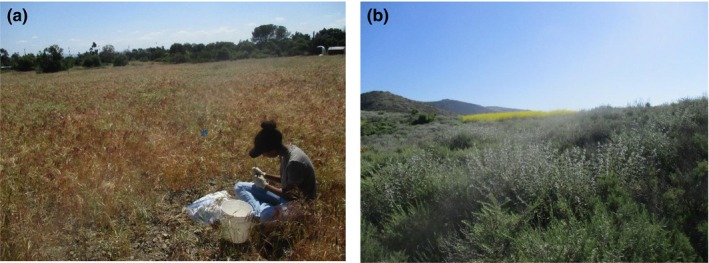
Pictures of habitat types studied: Anne Berhe (student researcher) collecting soil samples from the non‐native grassland at the Bernard Field Station (a), and native sage scrub in the foreground with non‐native forblands (yellow‐flowered mustard plants) in the background at the Santa Monica Mountain site (b)

To understand the effects of type‐conversion by common invasive annuals (non‐native grasses, *Bromus* spp.; and non‐native forbs, *B. nigra*; Figure 1) on soil nutrient storage in southern California, we expand on work by Caspi et al. (2018) by examining total C and total N, key soil properties, and microbial concentrations in sage scrub and invaded areas at nine sites across both spatial (i.e., coast to inland) and temporal (i.e., fall and spring) scales. We use the term “nutrient” to refer exclusively to soil C and N as opposed to other key nutrients (P, S, Ca, Mg, Na, K, etc.). Collecting soil from the uppermost mineral soil (A) horizon in native and non‐native habitat types at each site we: (a) quantified total C and total N; (b) determined the variation in key soil properties; and (c) compared bacterial and fungal abundances between habitat types and among sites. We hypothesized that type‐conversion to *Bromus‐*dominated non‐native grassland negatively impacts soil C and N storage, and sites with higher C and N storage would have soil properties more indicative of greater storage ability such as high cation exchange capacity (CEC) and clay content. We were also interested in assessing whether nutrient levels are similarly reduced by type‐conversion to non‐native mustard‐dominated forblands, and to what extent these two invasive landscapes differ in their relationship from native sage scrub. We predicted that bacterially dominated soils would have lower total C and N when compared to those of similar soil taxonomy that are fungal‐dominated. Because nutrient storage is determined by the balance of soil inputs and outputs, we were unsure how overall storage would fluctuate temporally. As spring is wetter than fall (Rundel, 2007), temporal shifts in nutrient storage are difficult to predict as we expect both an increase in plant inputs due to enhanced photosynthesis as well as an increase in microbial activity due to enhanced soil moisture. By surveying surface soil horizons along a representative southern California environmental gradient, we identify and model key factors that predict soil C and N storage throughout the region, and we further elucidate the impacts of contemporary large‐scale type‐conversion on these key ecosystem services.

## 2 MATERIALS AND METHODS

### 2.1 Study site

The sage scrub ecosystem is native to low‐lying areas of southern California and is composed largely of drought‐deciduous shrubs. Sage scrub in coastal areas is characterized by an increased abundance of evergreen shrubs such as *Rhus integrifolia* and* Malosma laurina*, while inland sites are characterized by drought‐tolerant species such as *Salvia apiana* and drought‐deciduous shrubs such as *Artemisia californica*(Rundel, 2007). This notable change in floristic assemblages is indicative of California's gradient of increasing continentality: Coastal vegetation is buffered from climatic extremes and sees increased humidity compared to inland vegetation experiencing heightened drought stress and broader temperature extremes (Bauer, 1936; Mooney & Zavaleta, 2016; Rundel, 2007). Further, topographic variation (slope angle and aspect), as well as differing soil taxonomies, is reflective of and contribute to this shift in plant communities across the gradient (Riordan & Rundel, 2014; Rundel, 2007). Regionally, substrate lithology in southern California varies from sites that may be dominated by sedimentary strata including sandstones and mudstones, to sites dominated by granitic alluvium (Graham, Schoeneberger, & Breiner, 2017; Soil Survey Staff, 2017). Soil taxonomy can also vary even within localized areas due to geologic factors (primarily lithologic variation due to stratigraphy and/or geologic structures).

**Figure 2:**
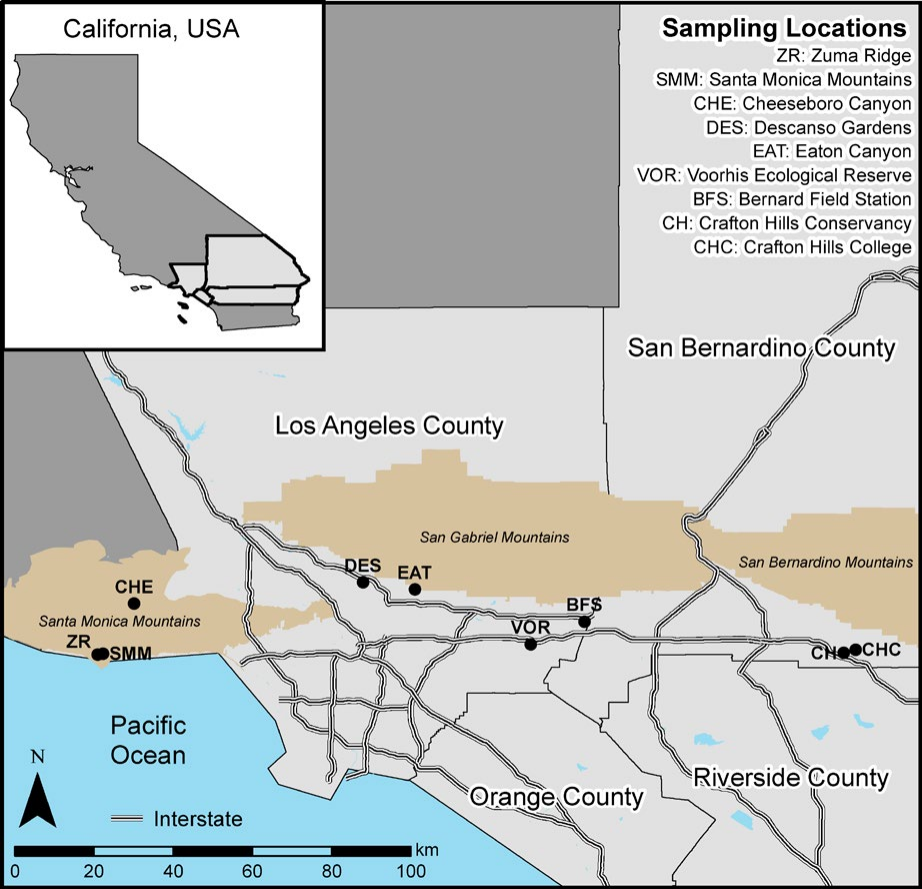
Location of nine sites sampled from along a coast to inland gradient in southern California in fall 2016 and spring 2017

We collected samples from nine sites in fall 2016 (October 14 through November 4) and spring 2017 (April 10 through May 2) along a coast to inland gradient from Los Angeles County to San Bernardino County (Figure 2). Many variables may be used to define a climatic or other environmental gradient (e.g., mean annual precipitation, mean annual temperature, average relative humidity, number of days without rainfall, maximum annual rainfall, annual insolation, etc.); however, no one variable perfectly defines the southern California coastal gradient; each has limitations for ecological significance. For convenience, we use straight‐line distance from the coast to broadly define the gradient in this study and to facilitate site‐to‐site comparison. We acknowledge that distance to the coast itself is not a control determining storage capacity, but instead serves as a proxy representing changes in atmospheric conditions as well as soil temperature and moisture from site to site. Zuma Ridge was the most coastal site, situated only 2 km from the Pacific Ocean while Crafton Hills College was furthest inland at 84 km from the coast. All sites contained an area of intact sage scrub and adjacent type‐converted non‐native annual grassland or non‐native forbland. Adjacent habitat types were within 300 m of each other to control for variation in soil properties or microclimate within sites. We defined sage scrub as habitat dominated mostly by *A. californica*and other native drought‐deciduous or evergreen woody shrubs while consisting of <10% cover of non‐native species by visual estimate. Non‐native annual habitats consisting of under 5% cover of native shrubs and dominated by either invasive *Bromus* spp. or invasive *B. nigra* were defined as non‐native grassland or non‐native forbland, respectively (see supporting Information Table S1 for a description of the plant compositions in each habitat at each site).

Inescapably, there is wide but representative topographic variation across these sites and soil taxonomy also differs across the gradient. Because soil properties may change with slope position (Pierson & Mulla, 1990), topographic differences in angle and aspect across our sampling locations may influence both soil conditions and nutrient storage. Caspi et al. (2018) suggest that soil surveys are a useful first approximation but cannot be relied upon to assess differences in soil characteristics below the 1:24,000 scale for which they are intended, especially with regard to precise accounting of soil C and N. As such, we collected our own measurements of soil pH, CEC, OM content, and soil texture (percent sand, silt, and clay), to compare soil properties across sites and between habitats. Given that sage scrub habitats exist in many areas with distinct parent materials, slope angles, and soil types in southern California, comparisons between unique soils are important to understand impacts of type‐conversion on soil C and N at the regional scope.

### 2.2 Sample collection and processing

We collected soil from both habitat types at each site from six sampling locations in the fall of 2016 (October–November) and spring of 2017 (April–May) to compare total C and total N, other soil properties, and microbial abundances. We gathered four different soil sample types from each sampling location. First, we obtained an intact soil clod from the mineral soil surface (A horizon) for calculating bulk density (Burt, 2004). We slightly modified the clod method used in Caspi et al. (2018) to decrease the number of times each soil clod is dipped in resin and to better account for hairnet weight. The final equation used is below (where *D* is density and *W* is weight).

$$D_{\text{soil}}=\frac{\left[\left(\left(W_{\text{dip1}}-\left(W_{\text{dip2}}-W_{\text{dip1}}\right)\right)-W_{\text{hairnet}}\right)\times \left(1-\left(\frac{W_{\text{dip2}}-W_{\text{oven}}}{W_{\text{dip2}}}\right)\right)\right]-W_{\text{rocks}}}{\text{Volume}}.$$

Second, we collected ~30 ml of loose soil from within the A horizon (the top ~10 cm) to analyze percent C and percent N using an Elementar vario MICRO cube elemental analyzer (Elementar Mt. Laurel, New Jersey). We froze samples at −20°C between sample collection and analysis. Third, we sent ~250 ml of soil from the A horizon at each sampling location in the spring only to Earthfort Laboratories (Corvallis, Oregon) immediately after collection to determine total and active bacteria and fungi. Direct enumeration through microscopy was used to quantify total bacteria and fungi (µg/g) (Babiuk & Paul, 1970; Ingham, 1995; Schnurer & Rosswall, 1982; Van Veen & Paul, 1979). Finally, in the spring season only, we collected a composite soil sample containing soil from each sampling location within a habitat type from each site to send to the UC Davis Analytical Laboratory (Davis, California) to measure soil properties (pH, CEC, OM, and soil texture).

### 2.3 Scaling and statistical analyses

We determined the quantity (g/m^2^) and the concentration (% per gram) of C and N in the uppermost mineral soil horizon (A horizon). Total C and total N quantities were scaled to report g C/m^2^ and g N/m^2^. While these values are reported as an area (m^2^), they represent the full depth of the observed soil A horizons, approximately 10 cm, to be representative of nutrient storage dynamics across the landscape. Quantity incorporates soil nutrient concentration with aggregate soil density and was calculated by multiplying C and N concentration by the clod density of the examined soil layer in order to exclude rocks. The soils we collected are not mapped by Soil Survey as containing CaCO_3,_ and all have acidic pH (Soil Survey Staff, 2017). Thus, we assume total C values represent only soil organic carbon—it is unlikely that any of the soils contain carbonate or other forms of soil inorganic carbon.

To examine how soil properties vary across the gradient, we conducted linear regressions to determine the relationship between each site's distance from the coast and pH, CEC, OM, and clay content. To compare soil properties between habitat types (native vs. invasive), we ran four paired *t* tests comparing pH, CEC, OM, and clay content.

To test for differences in nutrient concentration between habitats (native vs. invasive) and seasons (fall vs. spring), we ran four three‐factor univariate PERMANOVA tests using site, habitat, and season as factors. Because we assumed sites would significantly differ based on findings in Caspi et al. (2018), incorporating site as a factor allowed us to test for differences between habitats and seasons while controlling for differences among sites. We also ran four two‐factor univariate PERMANOVA tests using site and habitat as factors to test for differences in total and active bacteria and fungi between habitats. Because microbial data were only collected in the spring, season was not included as a factor. All tests were run using Euclidean distances in the program PRIMER‐E with the PERMANOVA+ add on (Anderson, Gorley, & Clarke, 2008). Five samples were excluded from C and N analyses (three from both C and N analyses, two from just C analyses) due to labeling errors or designation as outliers for having nutrient concentrations greater than commonly reported in the literature (Allison et al., 2005; Wolkovich, Lipson, Virginia, Cottingham, & Bolger, 2010), suggesting that materials from the O horizon were accidentally incorporated in the sample. Following significant PERMANOVA results for site × habitat interactions, we conducted pairwise comparisons to determine at which sites sage scrub and non‐native habitats differed significantly. We also ran post hoc PERMANOVAs excluding the two sites with non‐native forblands dominated by *B. nigra* (Santa Monica Mountains and Voorhis Ecological Reserve) to specifically test the effects of *Bromus*‐dominated type‐conversion on percent and total C and N.

To examine which variables best predicted C and N quantities across the gradient, we ran two backward stepwise regressions using the car package in R studio (Fox et al., 2017; R Core Team, 2017). We used pairwise scatterplots to choose variables that constrained redundancy while maintaining biologically important sets of conditions. For example, OM and clay content were, unsurprisingly, strongly correlated with CEC, thus representing similar soil property conditions. Elevation correlated with distance to the coast and was excluded from the analyses. Variables were considered collinear if the *r*‐value was >0.7. Microbial communities in soil are composed of microorganisms that exist in different physiological states: While active microorganisms are involved in biogeochemical transformations associated with nutrient turnover, those in dormant states do not contribute to ongoing microbial processes (Blagodatskaya & Kuzyakov, 2013). As such, we chose to incorporate active bacterial and fungal abundances rather than total abundances in the analyses to better represent the microbial communities actively contributing to ecosystem function. The final variables used included one categorical variable, habitat (sage scrub, non‐native grassland, or non‐native forbland), and five continuous variables, active bacteria, active fungi, distance to coast, pH, and CEC. Total C and total N quantities were log transformed to normalize the data. We determined which variables best predicted total C and N based on the Akaike information criterion (AIC).

## 3 RESULTS

### 3.1 Soil properties

Soil properties differed among sites as expected from USDA NRCS Soil Survey (Table 1). Inconsistencies between mapped soil classifications and observed soil properties likely result from the coarse (1:24,000 or broader) Soil Survey scales that mask small and localized soil variability. CEC and clay content decreased along the coast to inland gradient, being highest in the well‐developed Mollisols at coastal sites compared to the less‐developed Inceptisols and Entisols mapped at inland sites (CEC: *r* ^2^ = 0.460, slope = −0.245, *p* = 0.0020; clay content: *r* ^2^ = 0.623, slope = −0.361, *p* < 0.0001). OM content and pH did not change linearly along the coast to inland gradient (OM: *r* ^2^ = 0.193, slope = −0.024, *p* = 0.0689; pH: *r* ^2^ = 0.175, slope = −0.011, *p* = 0.0844).

**Table 1:** Soil properties for native sage scrub (CSS) and non‐native habitats (non‐native grassland [NNG] or non‐native forbland [NNF]) at nine sites along a coast to inland gradient in southern California

Site and habitat type	Distance to coast (km)	pH	Cation exchange capacity	Organic matter (%)	Soil texture		
					Sand (%)	Silt (%)	Clay (%)
Zuma Ridge CSS	2	6.73	43.8	6.36	29	26	45
Zuma Ridge NNG	2	7.94	40.2	4.49	35	21	44
Santa Monica Mountains CSS	2	5.21	23.2	5.80	35	34	32
Santa Monica Mountains NNF	2	6.66	42.7	7.59	32	24	44
Cheeseboro Canyon CSS	13	5.93	30.7	5.49	50	25	25
Cheeseboro Canyon NNG	13	6.90	32.0	4.59	48	25	27
Descanso Gardens CSS	34	5.54	18.7	4.75	61	25	14
Descanso Gardens NNG	34	6.54	20.1	4.62	63	24	13
Eaton Canyon CSS	41	5.56	11.8	2.65	88	6	6
Eaton Canyon NNG	41	6.29	13.3	2.33	83	11	7
Voorhis Ecological Reserve CSS	43	5.84	21.2	3.58	71	14	15
Voorhis Ecological Reserve NNF	43	4.83	23.6	3.27	67	17	16
Bernard Field Station CSS	55	5.11	9.5	3.50	82	11	7
Bernard Field Station NNG	55	5.77	8.4	1.62	85	9	6
Crafton Hills Conservancy CSS	82	5.42	16.9	5.04	68	21	11
Crafton Hills Conservancy NNG	82	6.38	17.6	3.21	65	21	14
Crafton Hills College CSS	84	5.09	22.6	6.58	67	23	10
Crafton Hills College NNG	84	6.45	19.2	2.59	73	18	9

Invasive habitats had higher pH compared to adjacent native sage scrub habitat, indicating that soils in sage scrub are generally more acidic (*t* = −3.33, *df* = 8, *p* = 0.0104). CEC, OM, and clay content did not differ between habitat types (CEC: *t* = −0.91; *df* = 8, *p* = 0.3894; OM: *t* = 1.96; *df* = 8; *p* = 0.0857; clay content: *t* = −1.16; *df* = 8; *p* = 0.2795).

### 3.2 Soil nutrients

**Figure 3:**
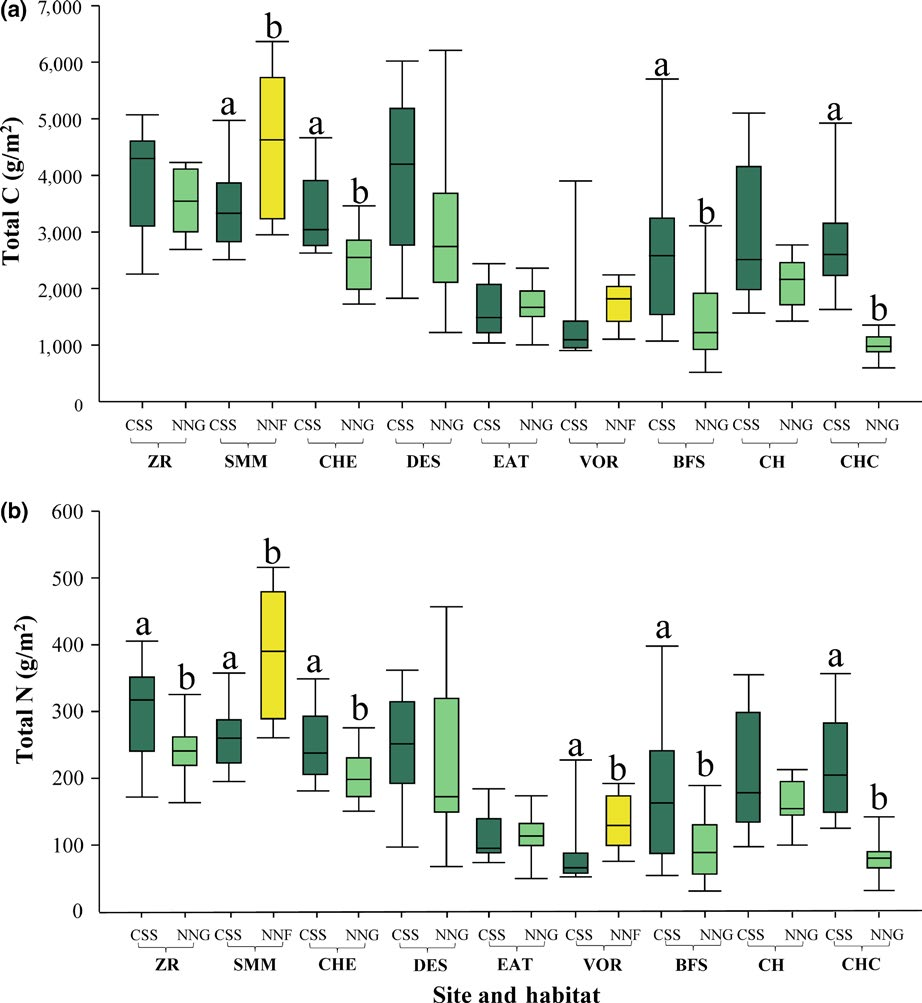
Total quantity (g/m^2^) of C (a) and N (b) in California sage scrub (CSS) and invasive habitats (non‐native grassland [NNG] or non‐native forbland [NNF]) at nine different sites along a coast to inland gradient in southern California. Sites marked with different letters differ following pairwise comparisons (*p* < 0.05). CSS is dark green, NNG is light green, and NNF is yellow

Native sage scrub habitat did not store more nutrients than non‐native habitat types (Table 2). However, for all measures of nutrient storage, there was a significant site × habitat interaction suggesting that the effect of type‐conversion on storage capacity is contingent upon site (Table 2). Pairwise comparisons examining differences in nutrient storage between habitats at each site revealed significant differences in total C between habitats at four sites (Figure 3). Of these sites, three containing *Bromus*‐dominated invasive habitats had higher C storage in the native sage scrub habitat compared to the adjacent non‐native grassland. In the site containing the non‐native forbland habitat, C storage was lower in the native habitat type. Pairwise comparisons for total N showed differences between habitat types at six sites: four sites containing *Bromus*‐dominated invasive habitats as well as the two sites with *Brassica*‐dominated invasive habitats (Figure 3). Similar to patterns in total C, total N was higher in native sage scrub habitat than invasive habitat at the four sites with non‐native grasslands, but lower in the native habitat at the two sites containing non‐native forblands.

**Table 2:** Results for PERMANOVA tests examining differences in nutrient storage in native sage scrub and non‐native habitats at nine different sites

Source	*df*	Percent C		Percent N		Total C		Total N	
		Pseudo‐*F*	*p*	Pseudo‐*F*	*p*	Pseudo‐*F*	*p*	Pseudo‐*F*	*p*
Site	8	25.2950	0.0001**	30.931	0.0001**	25.194	0.0001**	32.036	0.0001**
Habitat	1	4.2307	0.0704	1.7363	0.2260	3.0402	0.1219	0.9491	0.3562
Season	1	4.8605	0.0621	8.6897	0.0188*	0.0443	0.8282	1.4506	0.2637
Site × habitat	8	3.717	0.0005**	5.898	0.0001**	5.427	0.0001**	8.1608	0.0001**
Site × season	8	1.732	0.0924	2.5923	0.0101**	2.3237	0.0212*	3.4232	0.0010**
Habitat × season	1	0.0167	0.9011	2.5489E−6	0.999	0.2381	0.6320	0.1391	0.7197
Site × habitat × season	8	1.3697	0.2130	1.7315	0.0956	1.6015	0.1251	2.4104	0.0166*

* *p* = 0.05.

** *p* = 0.01.

Post hoc analyses that excluded sites with *Brassica*‐dominated non‐native forblands revealed that native sage scrub habitat stored more C and N than *Bromus*‐dominated non‐native grasslands (Table 3). Averaged among all sampling locations, native sage scrub habitat contained 2.88% C and 0.21% N whereas non‐native grassland soils contained 2.22% C and 0.16% N, over 22% less C and N. Non‐native forbland soils averaged 2.87% C and 0.24% N. Percent N was lower in the spring than in the fall, but seasons did not differ with regard to percent C, total C, or total N (Table 2).

**Table 3:** Results for PERMANOVA tests examining differences in nutrient storage in native sage scrub and non‐native grassland habitats (excluding non‐native forbland habitats) at seven different sites

Source	*df*	Percent C		Percent N		Total C		Total N	
		Pseudo‐*F*	*p*	Pseudo‐*F*	*p*	Pseudo‐*F*	*p*	Pseudo‐*F*	*p*
Site	6	17.7230	0.0001**	16.8890	0.0001**	18.1210	0.0001**	17.3270	0.0001**
Habitat	1	10.0840	0.0170*	7.7174	0.0302*	14.1190	0.0150*	10.6460	0.0180*
Season	1	4.4162	0.0778	7.3271	0.0372*	1.3975	0.2801	6.6607	0.0463*
Site × habitat	6	2.5176	0.0224*	3.2281	0.0050**	2.3414	0.0353*	2.5724	0.0198*
Site × season	6	1.9743	0.0738	2.8818	0.0109*	1.4764	0.1908	1.639	0.1405
Habitat × season	1	0.0677	0.8075	0.3042	0.5934	0.1297	0.7364	0.0703	0.7946
Site × habitat × season	6	1.4015	0.2215	1.5220	0.1741	0.8289	0.5492	1.2383	0.2913

* *p* = 0.05.

** *p* = 0.01.

### 3.3 Microbial abundance

**Figure 4:**
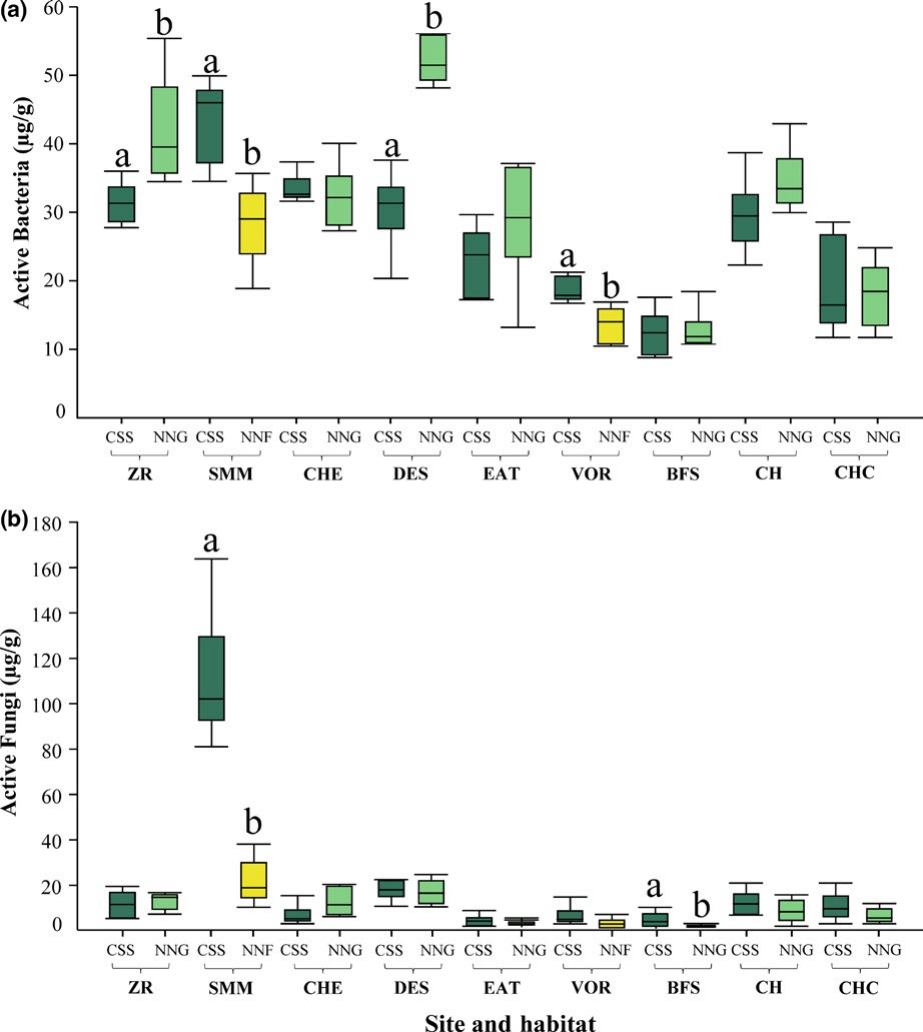
Active bacterial abundance (a) and active fungal abundance (b) in California sage scrub (CSS) and invasive habitats (non‐native grassland [NNG] or non‐native forbland [NNF]) at nine different sites along a coast to inland gradient in southern California. Sites marked with different letters differ following pairwise comparisons (*p* < 0.05). CSS is dark green, NNG is light green, and NNF is yellow

Habitat alone was not a significant factor driving microbial abundances, but a significant site and habitat interaction suggests that the impact of type‐conversion on microbial abundances is dependent upon site location (Table 4). Pairwise comparisons revealed significant differences between habitat types for active bacteria at four sites, two containing non‐native grasslands and two containing non‐native forblands (Figure 4). Active bacterial abundance was lower in non‐native forblands compared to adjacent native sage scrub habitat types, but higher in non‐native grasslands. Active fungal counts were generally very low except in the sage scrub habitat of the Santa Monica Mountains in which abundance was at least five times greater than any habitat in any other site (Figure 4). Pairwise comparisons for active fungal abundance only revealed significant differences between habitats at two sites, one containing non‐native forbland and the other containing non‐native grassland. At both these sites, fungal abundance was higher in the native sage scrub habitat.

**Table 4:** Results for PERMANOVA tests examining differences in microbial abundance in native sage scrub and non‐native habitats at nine different sites

Source	*df*	Active bacteria		Active fungi		Total bacteria		Total fungi	
		Pseudo‐*F*	*p*	Pseudo‐*F*	*p*	Pseudo‐*F*	*p*	Pseudo‐*F*	*p*
Site	8	48.5630	0.0001**	67.6740	0.0001**	36.8070	0.0001**	43.0210	0.0001**
Habitat	1	0.4580	0.5117	1.2608	0.4747	3.7959	0.0858	0.9158	0.3745
Site × habitat	8	12.4180	0.0001**	37.7780	0.0001**	1.7018	0.1070	5.0354	0.0002**

* *p* = 0.05.

** *p* = 0.01.

### 3.4 Multiple regression

**Figure 5:**
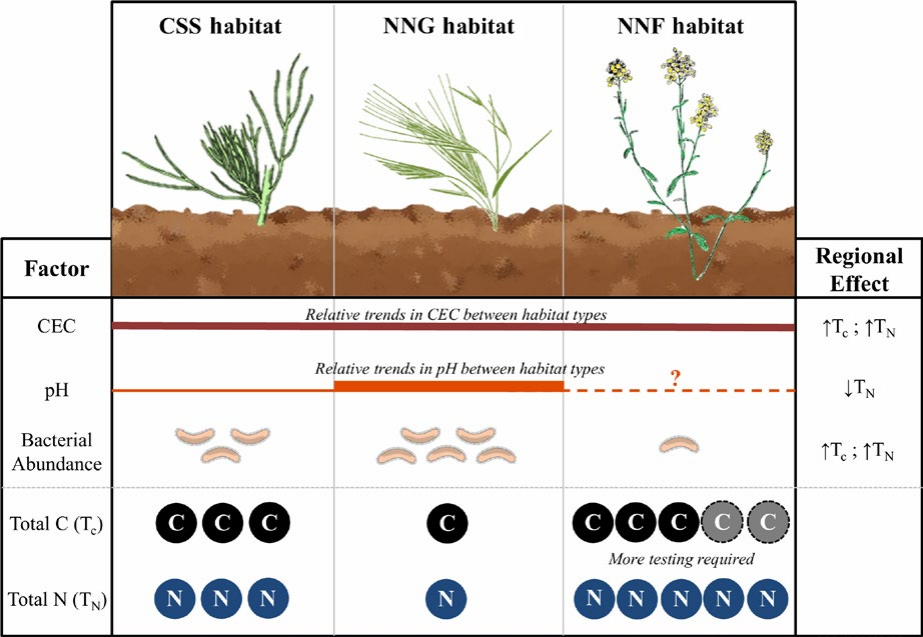
Conceptual model indicating differences in cation exchange capacity (CEC), pH, bacterial abundance, total C, and total N between habitats within any theoretical site in southern California containing two or more adjacent habitat types. The column labeled “Regional Effect” indicates the impact of each factor on total C or total N regionally, across a number of sites along a coast to inland gradient as predicted from the multiple regression. For CEC and pH, differences in line thickness represent relative relationships among those properties. The dashed line for pH indicates that further testing is required. Individual bacteria, circles labeled “C,” and circles labeled “N” represent relative concentrations of bacteria, total C, and total N, respectively. Dotted lines around circles labeled “C” indicate that patterns require further testing for confirmation

The variables selected for total C and total N differed (Tables 5 and 6). The strongest predictors of total C were CEC (ΔAIC = +22.34), active bacteria (ΔAIC = +15.45), and habitat type (ΔAIC = +11.61; Multiple *R* ^2 ^= 0.4983, *p* < 0.001; Table 5). For total N, CEC (ΔAIC = +51.33), active bacteria (ΔAIC = +15.21), and pH (ΔAIC = +11.41) were the best predictors of storage (Multiple *R* ^2^ = 0.5089; *p* < 0.001; Table 6). The direction of these relationships was consistent between total C and total N: Nutrient storage was positively correlated with CEC and active bacterial abundance. Total C negatively correlated with non‐native grassland habitat type, but non‐native forbland was not selected as a predictor of total C storage. Lower pH was associated with higher total N, suggesting greater N storage in more acidic soils. Distance to coast and active fungi were not selected as predictors of total C (distance: ΔAIC = −1.97; active fungi: ΔAIC = −1.39) or total N (distance: ΔAIC = −1.64; active fungi: ΔAIC = −0.99). A schematic representing the main effects of type‐conversion as well as the strongest predictors of regional nutrient storage was constructed to provide theoretical context for understanding how the factors associated with nutrient storage may interact with one another (Figure 5).

**Table 5:** Drivers of total C along a coast to inland gradient in southern California based on AIC selection

Coefficient	Estimate	*SE*	*t*	*p*
Intercept	6.8901	0.1155	59.643	<2.00E−16**
Habitat (non‐native forbland)	−0.0232	0.1411	−0.164	0.8699
Habitat (non‐native grassland)	−0.3319	0.0836	−3.972	0.0001**
Active Bacteria	0.0164	0.0037	4.251	4.720E−5**
Cation exchange capacity	0.0225	0.0044	5.104	1.540E−6**

Notes

Residual standard error = 0.3943; *df* = 102, Multiple *R* ^2^ = 0.4983; Adjusted *R* ^2 ^= 0.4786; *F*‐statistic = 25.32; *p* = 1.401E‐14.

**Table 6:** Drivers of total N along a coast to inland gradient in southern California based on Akaike information criterion selection

Coefficient	Estimate	*SE*	*t*	*p*
Intercept	5.2270	0.3456	15.125	<2.00E−16**
pH	−0.2572	0.0694	−3.709	0.0003**
Active Bacteria	0.0166	0.0039	4.239	4.91E−05**
Cation exchange capacity	0.0393	0.0048	8.157	8.81E−13**

Notes

Residual standard error = 0.4198; *df* = 103; Multiple *R* ^2^ = 0.5089; Adjusted *R* ^2^ = 0.4946; *F*‐statistic = 35.57; *p* = 7.341E‐16.

## 4 DISCUSSION

### 4.1 Local effects of invasion

Our study emphasizes the importance of recognizing and incorporating the identity of the invader when determining the impact type‐conversion has on regional nutrient storage. While C and N concentrations were over 22% higher in sage scrub than non‐native grassland habitats, these same differences were not observed between sage scrub and non‐native forbland habitats. Further, pairwise comparisons between adjacent sage scrub and non‐native forbland habitats revealed greater N storage in non‐native forblands compared to sage scrub at two sites and greater C storage in non‐native forblands at one site. These findings indicate that nutrient storage may be higher in non‐native forblands compared to adjacent sage scrub habitats, but further examination focusing on *Brassica* spp. invasions is required. Specifically, further testing through manipulative studies that add *Brassica*spp. to experimentally controlled sites is needed to confidently ascribe causation to invasive forbs in altering soil nutrient storage patterns. Our findings demonstrate that invasion does not impact soil C and N storage unidirectionally: The composition of the invading community plays a key role in determining storage patterns. Categorizing habitat as native or invasive in our analyses masked the negative impact of *Bromus*‐dominated grasslands on storage capacity. Only in post hoc analyses, in which non‐native forblands were excluded, was this effect apparent. Commonly, studies focusing on sage scrub ecosystems often refer to invasive annual cover as “grass cover” (Talluto & Suding, 2008) or “grassland” (Caspi et al., 2018), even when non‐native forbs, such as *Brassica* spp., are the dominant or codominant plant type. Additionally, habitat may be misclassified as sage scrub even when the majority of cover (≥75%) is composed of non‐native species (Matsuda et al., 2011; Suarez et al., 1998). Our results indicate the importance of not only differentiating between native sage scrub and invasive habitat, but also of classifying invasive habitats dominated by grasses as distinct from those dominated by non‐native forbs.

In our study, soil under *Brassica‐*dominated non‐native forbland habitat contained significantly fewer microbes than adjacent soil under native sage scrub habitat, suggesting that invasion by mustards and grasses may differentially influence microbial communities and overall nutrient storage patterns. Shifts in microbial communities are likely a result of different qualities of OM returned to the soil by plants with different ecophysiological attributes or suites of traits (Díaz et al., 2004; Wardle, 2006). Toxins emitted into the soil by stands of *Brassica*species specifically are known to degrade mycorrhizal fungi symbioses and inhibit spore germination (Pakpour & Klironomos, 2015; Schreiner & Koide, 1993), therefore altering microbial communities in the soil. Inconsistent with our hypothesis, fungal abundances were lower than bacterial abundances at all but one sampling location, suggesting that differences in nutrient storage between habitat types were likely not determined by differing fungal:bacterial ratios. On the other hand, several pairwise comparisons revealed significant differences in active bacterial abundances suggesting that, within sites, sage scrub habitats have lower bacterial counts than adjacent non‐native grassland, but greater bacterial concentration than adjacent non‐native forblands. This pattern in microbial abundances in combination with observed nutrient storage patterns suggests that habitats with higher bacterial counts are associated with lower nutrient storage than habitats with lower bacterial counts. Bacterially dominated soils are characterized by rapid plant litter decomposition and nutrient mineralization rates which result in reduced C storage (Manning, 2012; Wardle, 2002). This pattern of enhanced microbial decomposition in surface horizons beneath invasive grasslands compared to adjacent soils under native vegetation has occurred in other regions (Norton, Monaco, Norton, Johnson, & Jones, 2004). However, the exact mechanism by which different non‐native plant communities alter and maintain unique microbial communities is not yet well understood (Caspi et al., 2018; Wardle, 2006).

The quality of substrates often plays a key role in determining decomposition rates (Chapin, Matson, & Mooney, 2002), influencing C and N cycling patterns. Plants with higher quality litters (e.g., those with low C:N ratios) decompose more quickly (Chapin et al., 2002; Enriquez, Duarte, & Sand‐Jensen, 1993; Zhang, Hui, Luo, & Zhou, 2008). The C:N ratio of *Bromus*species is greater than that of native sage scrub species (Dipman & Meyer, 2019; Wolkovich et al., 2010), suggesting that decomposition may be slower (and bacterial activity lower) in non‐native grassland compared to sage scrub. However, in semiarid regions, UV radiation reaching the soil can facilitate photochemical degradation of litter compounds, increasing the biodegradability of litter (Foereid, Bellarby, Meier‐Augenstein, & Kemp, 2010). Studies in southern California and other semiarid regions have demonstrated UV to be a primary determinant of decomposition rates (Austin & Vivanco, 2006; Brandt, King, Hobbie, Milchunas, & Sinsabaugh, 2010; Day, Zhang, & Ruhland, 2007; Dipman & Meyer, 2019), and the effect of UV may be stronger in non‐native grassland than in sage scrub habitat (Dipman & Meyer, 2019). As such, different chemical compositions of plant litter may not be the only determinants of differences in decomposition rates and associated active bacterial abundances between sage scrub and non‐native grassland.

Though soil biota are likely to vary temporally due to the seasonal variation in the quantity and timing of inputs to the soil by plant species (Wardle, 2002), we did not collect data for microbial abundances in the fall. We did, however, observe seasonal changes in percent N with soil N concentration being lower in the spring season than in the fall. The seasonality of nitrogen mineralization is frequently different from the seasonality of nitrogen uptake by the plant community (Chapin et al., 2002). Soil microbes may continue to mineralize nitrogen during the fall season in which plants are dormant or dead and productivity is low, leading to an accumulation of available nitrogen that plants likely uptake when they become active (Chapin et al., 2002). A spring increase in plant N uptake coupled with an active fall microbial community could explain this seasonal reduction in percent N. Future studies should investigate whether microbial abundances change seasonally to provide greater insight into the mechanisms behind seasonal fluctuations in soil N concentration.

### 4.2 Regional patterns

Of all factors tested, CEC was the best predictor of total C and total N storage across the gradient. As such, local soil‐geomorphic factors may be the best predictors for determining storage capacity. Following expected patterns based on regional geology, CEC was highest at the coast, lowest in the middle of the gradient, and increased again slightly at the most inland sites. A higher CEC is reflective of more phyllosilicate minerals and soil organic content among other factors. This increases as well as reflects nutrient storage capacity since clay minerals and soil OM both increase the water‐holding capacity of soils. When combined with finer soil textures (greater abundance of phyllosilicate minerals), increased moisture periodically reduces the oxygen supply and the decomposition of OM by binding OM to clays (Chapin, 1997). As such, CEC is a particularly informative variable for modeling soil nutrient storage capacity throughout the region.

Active bacterial abundance was positively correlated to C and N storage. While within‐site habitat differences suggest higher bacterial counts result in decreased nutrient storage, the multiple regression highlights how, on a regional scale, sites—not habitats—with greater microbial abundances may be indicative of greater storage ability. Most bacteria become inactive when substrates are exhausted (Chapin, 1997). Lower bacterial activity as a predictor of decreased C and N storage may potentially be indicative of low resource availability, whereas increased bacterial activity may indicate increased C and N availability. These relationships require further investigation with regard to the controlling mechanisms, and examining to what extent OM inputs or microbial respiration rates determine soil nutrient storage in the region is an active line of inquiry. Ultimately, microbial activity is indicative of differences in the quantity, quality, and timing of OM inputs to the soil (Wardle, 2002).

As expected, habitat type was a predictor of total C, with decreased storage associated with non‐native grasslands. However, habitat was not selected as a variable predicting total N patterns. Instead, the model indicates that soil pH is a better predictor for total N, with higher pH correlated to lower overall N storage. Soil pH, which was lower in non‐native habitats, is a known variable driving and reflecting N patterns (Smithwick, Kashian, Ryan, & Turner, 2009). Many factors, such as changes in litter quality and high rates of ammonium uptake, can acidify soils (Ehrenfeld, 2003). Conversely, increases in pH may be associated with increased cation concentration in litter or preferential uptake of nitrate (Ehrenfeld, 2003). Regardless of the mechanism changing acidity, lower pH is commonly linked to low decomposition rates (Chapin et al., 2002), suggesting that nutrient turnover may be quicker with less acidic soils or in soils under *Bromus* grasslands in this study. Studies determining net nitrification often report lower rates as pH declines (Haynes, 1996), with the lower limit being around a pH of 4.5 (Sahrawat, 1982). Hawkes et al. (2005) found that gross nitrification doubled under invasive *Bromus* grasses compared to native species in an annual grassland in northern California. The ecological implications associated with this increase in nitrification are important: Enhanced nitrification increases the possibility of N loss through denitrification or leaching (De Boer, Hundscheid, Schotman, Troelstra, & Laanbroek, 1993; Hawkes et al., 2005), perhaps explaining lower total N found in non‐native grassland habitats in our study. Additionally, an increase in nitrification can result in greater plant‐available N for non‐native species that preferentially uptake nitrate over ammonium, theoretically contributing to a positive feedback cycle that promotes non‐native grass fitness and dominance over native species (Hawkes et al., 2005; Sperry, Belnap, & Evans, 2006). Ultimately, as non‐native grass invasion causes changes in both soil pH and microbial assemblages (Caspi et al., 2018; Sigüenza et al., 2006), N cycling patterns are altered. This type of plant‐microbe interaction, in which invading *Bromus* alters microbial community composition and nutrient availability patterns, is one possible pathway through which it has been able to successfully establish in sage scrub habitats (Norton, Monaco, & Norton, 2007; Sperry et al., 2006).

Nitrogen deposition, though unexplored in this study, poses serious implications with regard to ecosystem function (Padgett, Allen, Bytnerowicz, & Minich, 1999), as up to 45 kg N per hectare per year is deposited throughout the Los Angeles Air Basin (Bytnerowicz & Fenn, 1996; Fenn et al., 2003). Increased N enrichment has been linked to shifts toward bacterial‐based microbial communities as higher available N favors rapid decomposition and acceleration of nutrient turnover by bacteria (Manning, 2012). Enhanced decomposition could result not only in the long‐term depletion of C, but also in N loss through leaching (Hawkes et al., 2005; Manning, 2012; Norton et al., 2004). The possible impact of N deposition on regional nutrient storage should be further explored and potentially incorporated into future predictive models.

Our results demonstrate that *Bromus‐*dominated type‐conversion in sage scrub ecosystems reduces nutrient storage capacity. However, invasion by non‐native forbs (*B. nigra*) does not follow these same patterns and may increase soil nutrient storage. Scaling our measurement up to emphasize the role grass invasion plays in nutrient storage, we found that sage scrub habitats stored over 940 t C km^−2^ and over 60 t N km^−2^ more than non‐native grassland habitats. Talluto and Suding (2008) estimated that sage scrub habitat decreased by 49% over a 76‐year period from the 1930s. Given the scale of nutrient storage loss in a single square kilometer, this poses serious implications with regard to overall regional nutrient storage capacity. Our results provide key evidence that restoration and conservation efforts that restore and protect sage scrub will enhance nutrient storage, an increasingly important ecosystem service. Additionally, we provide a conceptual model for understanding the variables associated with contemporary and future nutrient storage in surface soils of southern California. Modeling regional nutrient storage requires distinct classification of habitat type as well as the consideration of soil CEC, pH, and bacterial concentration as predictors of storage capacity.

## CONFLICT OF INTEREST

The authors declare no conflicts of interest.

## AUTHOR CONTRIBUTION

Conceptualization, W.M.M. and C.R.R.; methodology, T.C., C.R.R., and W.M.M.; investigation, T.C., L.A.H., A.E.S., J.A.L., and W.M.M.; formal analyses, T.C., and W.M.M.; data curation, W.M.M. and T.C.; writing—original draft preparation, T.C., W.M.M., and C.R.R.; writing—review and editing, T.C., L.A.H., A.E.S., J.A.L., C.R.R., and W.M.M.; visualization, T.C., L.A.H., A.E.S., and J.A.L.; supervision, C.R.R. and W.M.M.; project administration, W.M.M.; funding acquisition, W.M.M.

## Data Availability

The dataset is available in the Knowledge Network for Biocomplexity repository (https://knb.ecoinformatics.org/view/doi:10.5063/F1GX48TP).
