# Supplementary material for: Impacts of invasive annuals on soil carbon and nitrogen storage in southern California depend on the identity of the invader
Source: Ecol Evol. 2019 Apr 1;9(8):4980–93. doi: 10.1002/ece3.5104 (PMC6476773; doi:10.1002/ece3.5104)
Supplement: Supplementary file 1 [file ECE3-9-4980-s001.docx]

| Site | Dominant Species | Other Species |
| --- | --- | --- |
|  |  |  |
| Zuma Ridge CSS | *Artemesia californica* & *Salvia leucophylla* | *Elymus* spp. |
| Zuma Ridge NNG | *Avena* spp*.* & *Bromus* spp. | *Brassica* spp., *Centaurea melitensis*, |
| Santa Monica Mountains CSS | *A. californica* | *Malosma laurina*,  *Mimulus aurantiacus, S. leucophylla* |
| Santa Monica Mountains NNF | *Brassica* spp. | *C. melitensis*, *Silybum marianum* |
| Cheeseboro Canyon CSS | *A. californica* & *S. leucophylla* | *Quercus* spp. |
| Cheeseboro Canyon NNG | *Bromus* spp. | *Brassica* spp*.,* *Carduus pycnocephalus* |
| Descanso Gardens CSS | *Salvia mellifera* | *A. californica*, *Brassica* spp., *M. laurina*, Pseudognaphalium californicum, *Hypericum scouleri* |
| Descanso Gardens NNG | *Bromus* spp. | *C. pycnocephalus*, *Dichelostemma capitatum* |
| Eaton Canyon CSS | *A. californica* | *Brassica* spp*.*,  *Bromus* spp., *Camissoniopsus* spp*.*, *C. melitensis* *Cryptantha* spp., Eriogonum fasciculatum, *Phacelia* spp. |
| Eaton Canyon NNG | *Bromus* diandrus | *Acmispon glaber*, *C. melitensis*, *Phacelia distans* |
| Voorhis Ecological Reserve CSS | *A. californica* | *Brassica* spp., *C. melitensis*, Eriogonum fasciculatum, Marah macrocarpa, *M. aurantiacus, Solalum* *umbellieferum* |
| Voorhis Ecological Reserve NNF | *Brassica* spp. & *Centaurea melitensis* | *A. californica*, *A. glaber*, *Pennisetum setaceum*, *Opuntia* spp. |
| Bernard Field Station CSS | *A. californica* | *Ericameria pinifolia*, *Erodictyion californicum*, *Lepidospartum squamatum, M. laurina*, *Penstemen spectabilis.*, *Opuntia* spp.,  *Salvia apiana, Sambucus nigra.*, *Toxicodendron diversilobum*, Rhamnus crocea |
| Bernard Field Station NNG | *Bromus* spp. | *Avena* spp*.*, *Brassica* spp., Croton setiger, *Erodium* spp., *S. nigra* |
| Crafton Hills Conservancy CSS | *A. californica* & *Eriogonum fasciculatum* | *Amsinckia* menziesii, *Brassica* spp., *Bromus* spp*.*, *Erodium* spp., *P. disdans* |
| Crafton Hills Conservancy NNG | *Bromus* spp*.* | *Avena* spp*.*, *Brassica* spp., *Erodium* spp. |
| Crafton Hills College CSS | *Ceanothus* spp. | *Adenostoma fasciculatum*, *Brassica* spp*.*, *Bromus* spp*.*, *C. melitensis, Cryptantha* spp*.*, *Ericameria* spp*.*, *Eschscholzia californica*, *Madieae* spp*.*, *Phacelia* spp., *S. mellifera*, |
| Crafton Hills College NNG | *Bromus* spp. | *Avena* spp*.,* *Brassica* spp, *E. californica, Erodium* spp., *Madieae* spp*.*, *Phacelia* spp. |

Table S1: Plant species observed in California sage scrub (CSS) and invasive habitats (non-native grassland (NNG) or non-native forbland (NNF)) at nine sites along a coast to inland gradient in southern California.
